# Special Issue “Advances in Antiviral Agents against SARS-CoV-2 and Its Variants”

**DOI:** 10.3390/v15091905

**Published:** 2023-09-10

**Authors:** Francesca Esposito, Rolando Cannalire

**Affiliations:** 1 Department of Life and Environmental Sciences, University of Cagliari, Cittadella Universitaria SS554, 09042 Monserrato, Italy; 2 Department of Pharmacy, University of Naples Federico II, via D. Montesano 49, 80131 Naples, Italy; rolando.cannalire@unina.it

Severe acute respiratory syndrome coronavirus 2 (SARS-CoV-2), with 770 million reported cases and around 7 million deaths, represents the worst pandemic in the last 100 years. Coronaviruses are a family of viruses that cause disease in mammals and birds, and, thanks to its airborne transmission, in humans cause infections of the respiratory tract that can be fatal.

With the advent of the pandemic, to find effective treatments capable of containing and limiting infections at an unprecedented speed, the scientific community has intensified its efforts to identify treatments for COVID-19, with enormous results obtained in molecular virology, biological screening platforms and drug discovery. 

Valid drugs have been found and various vaccines validated, which have proved effective in containing the spread of the pandemic. Despite the administered doses of vaccines, most of the population has been infected with the virus, often showing mild or severe symptoms of the infection. Although a third vaccine dose was needed to target emerging viral variants, continued progress needs to be made with antiviral agents against SARS-CoV-2 and its variants for any infections caused by emerging and re-emerging viruses.

This Special Issue, focusing on “Advances in Antiviral Agents against SARS-CoV-2 and its Variants” and the development of new therapies, presents nine peer-reviewed reports that describe research related to these areas of study.

During the COVID-19 pandemic, drug reuse has been an effective strategy to get quick responses to medical emergencies. Based on previous data on methotrexate (MTX), we evaluated the antiviral activity of several DHFR inhibitors in two cell lines. Interestingly, pralatrexate and trimetrexate showed superior effects in counteracting viral infection compared to other DHFR inhibitors. Our results indicate that their highest activity is due to their polypharmacological and pleiotropic profile. These compounds can then potentially give a clinical advantage in the management of SARS-CoV-2 infection in patients already treated with this class of drugs [1].

Remdesivir (RDV), an antiviral agent with in vitro efficacy against coronaviruses, is a potent and safe treatment, as suggested by numerous in vitro and in vivo studies and clinical trials. Emerging real-world data has confirmed its effectiveness, and datasets currently exist evaluating its efficacy and safety against SARS-CoV-2 infections in various clinical scenarios. Remdesivir increases the chances of recovery, reduces progression to severe disease, lowers mortality rates and shows beneficial post-hospitalization results, especially when used in the early stages of the disease [2].

One study investigates the activity of the remdesivir–nirmatrelvir combination against Severe Acute Respiratory Syndrome Coronavirus-2 (SARS-CoV-2) and reports a case of Coronavirus Disease 2019 (COVID-19) cured with this combination, showing that the remdesivir–nirmatrelvir combination has synergic activity in vitro. This combination may have a role in immunosuppressed patients with severe COVID-19 and prolonged viral shedding [3].

An inactivated vaccine administered intramuscularly is one of the most commonly used coronavirus disease 2019 (COVID-19) vaccines in developing regions. One study hypothesized that intranasal boosting after intramuscular priming would provide a broader level of protection, demonstrating that one or two intranasal boosts with the Fc-linked trimeric receptor binding domain of wild-type SARS-CoV-2 can induce significantly higher serum neutralizing antibodies against wild-type SARS-CoV-2 and Omicron subvariants, including BA.5.2 and XBB.1 [4].

While protective vaccines are available, concerns remain as new variants of the virus continue to appear. CRISPR-based gene editing approaches offer an attractive therapeutic strategy as CRISPR-RNA (crRNA) can be rapidly adapted to accommodate a new viral genome sequence. The use of an RNA-targeted CRISPR-Cas13d system to attach highly conserved sequences in the viral RNA genome represents a means of preparing for future zoonotic outbreaks of other coronaviruses, thus demonstrating the breadth of this antiviral strategy [5].

Realizing that the increasing variations of SARS-CoV-2 significantly impact the efficacy of antiviral therapies and vaccines, one review summarizes the appearance and attributes of SARS-CoV-2 variants for future perspectives in drug design, providing up-to-date insights for the development of agent therapies that target variants. The Omicron variant is among the most mutated forms; its strong transmissibility and immune resistance have attracted international concern. Most of the mutation sites currently studied are in the BCOV_S1_CTD of the S protein. Despite this, several obstacles remain, such as the development of effective vaccinations and drug treatments for emerging mutants of SARS-CoV-2 strains [6].

SARS-CoV-2 canonically uses clathrin-mediated endocytosis (CME) and several other endocytic mechanisms to invade airway epithelial cells. Endocytic inhibitors, particularly those targeting CME-related proteins, have been identified as promising antiviral drugs. Currently these inhibitors are ambiguously classified as chemical, pharmaceutical or natural inhibitors. A new mechanistic mechanism-based classification of endocytosis inhibitors, where they are segregated into four distinct classes. Excluding antiviral drugs designed to halt SARS-CoV-2 replication, other drugs, either approved by the FDA or suggested through basic research, could be systematically assigned to one of these classes [7].

To reduce the risks of airborne transmission of this powerful pathogen, an inactivation method based on the propagation of electromagnetic waves in the area to be sanitized has been devised. Conditions were optimized in a controlled laboratory environment by mimicking the natural transmission of an airborne virus and consistently achieving a 90% (ten-fold) reduction in infectivity after a short treatment using radio frequency (RF) wave emission with a potency level that is safe for people according to most regulatory agencies, including those in Europe, the United States, and Japan. It has been demonstrated, for the first time, that SARS-CoV-2 is inactivated through the emission of RF waves under conditions compatible with the presence of humans and animals [8].

A study has outlined and highlighted key elements of recent advances in non-thermal biocompatible plasma (NBP) technology for antiviral applications. Documents on NBP virus inactivation were searched in the PubMed ePubs, Scopus, and Web of Science databases. Relevant data and information were collected in order to establish a mechanism for NBP-based viral inactivation. NBP was developed as a new, effective, and safe strategy for viral inactivation. Scientists are developing NBP technology solutions to assist the medical community in addressing the current COVID-19 outbreak. NBP is expected to be the most promising strategy to fight COVID-19 and other viruses in the future [9].

The broad scope and importance of these works highlight the importance of applying a broad range of research tools to inhibit viral replication.

Future success will require accurate and rapid diagnosis globally, a better understanding of viral transmission patterns, the availability of increasingly effective broad-spectrum vaccines including variants, as well as the development of effective patient treatment strategies.
